# Risk factors for carbapenem-resistant *K. pneumoniae* bloodstream infection and predictors of mortality in Chinese paediatric patients

**DOI:** 10.1186/s12879-018-3160-3

**Published:** 2018-05-31

**Authors:** Ye Zhang, Ling-Yun Guo, Wen-Qi Song, Yan Wang, Fang Dong, Gang Liu

**Affiliations:** 1 Department of Infectious Disease, Beijing Children’s Hospital, Capital Medical University, No. 56 Nanlishi Road, Xi Cheng District, Beijing, 100045 People’s Republic of China; 2 Department of Laboratory Medicine, Beijing Children’s Hospital, Capital Medical University, No. 56 Nanlishi Road, Xi Cheng District, Beijing, 100045 People’s Republic of China

**Keywords:** Carbapenem-resistant *K. pneumoniae*, Bloodstream infection, Risk factor, Children

## Abstract

**Background:**

Bloodstream infections (BSI) caused by carbapenem-resistant *K. pneumoniae* (CRKP) are associated with high rates of morbidity and mortality. Early identification of patients at highest risk is very important. The aim of this study was to describe the clinical characteristics and mortality of *K. pneumoniae* BSI and to identify risk factors associated with CRKP BSI among paediatric patients.

**Methods:**

From January 2011 to December 2014, a retrospective case-control study was conducted at Beijing Children’s Hospital, China. Risk factors for CRKP BSI and for *K. pneumoniae* BSI-related death were evaluated. Patients with BSI caused by *K. pneumoniae* were identified from the microbiology laboratory database. Data regarding demographic, microbiological and clinical characteristics, therapy and outcome were collected from the medical records.

**Results:**

A total of 138 patients with *K. pneumoniae* BSI were enrolled, including 54 patients with CRKP BSI and 84 patients with carbapenem-susceptible *K. pneumoniae* (CSKP) BSI. Most of the BSI (114; 82.6%) were healthcare-associated, while the rest (24; 17.4%) were community-acquired. Hematologic malignancies (odds ratio (OR):4.712, [95% CI: 2.181–10.180], *P* <  0.001) and previous cephalosporin administration (OR: 3.427, [95% CI: 1.513–7.766], *P* = 0.003) were found to be associated with the development of CRKP BSI. 28-day mortality of *K. pneumoniae* BSI was 8.7%. Mechanical ventilation (OR:9.502, [95% CI: 2.098–43.033], *P* = 0.003), septic shock (OR:6.418, [95% CI: 1.342–30.686], *P* = 0.020), and isolation of CRKP (OR:9.171, [95% CI: 1.546–54.416], *P* = 0.015) were independent risk factors for 28-day mortality of *K. pneumoniae* BSI.

**Conclusion:**

Hematologic malignancies and previous cephalosporin administration were associated with the development of CRKP BSI, while mechanical ventilation, septic shock and CRKP infection were independent mortality predictors for *K. pneumoniae* BSI. More attention should be paid to CRKP BSI in the paediatric population.

## Background

During the last decade, carbapenem resistant *K. pneumoniae* (CRKP) has spread worldwide, including to China [1, 2]. This organism is rapidly becoming a major threat to public health because of the limited therapeutic options and the high morbidity and mortality. Previous studies found that mortality rates ranged from 30 to 50% for infections caused by CRKP [3–6]. In China, this pathogen became endemic in many regions; the data from CHINET (an antimicrobial resistance surveillance network in China) surveillance regarding bacterial resistance demonstrated a marked increasing trend of CRKP between 2005 and 2014, with the rate rising from 2.4 to 13.4% [7].

CRKP can cause many types of infections, including pneumonia, urinary tract infections, bloodstream and intra-abdominal infections [8, 9]. Bloodstream infection (BSI) is the most important infection, with a high risk of mortality [10]. At its worst, with broad-spectrum antibiotic resistance, treatment options for BSI caused by CRKP are very limited [11, 12].

Because there are fewer therapeutic options in children [13, 14], the treatment of CRKP BSI is a real challenge for the paediatrician. The keys to success in preventing and treating CRKP infections are the implementation of infection control measures and early detection of patients at highest risk [14]. In this context, recognizing the risk factors associated with the development of CRKP BSI is of great importance when considering treatment options. Many studies have explored risk factors associated with carbapenem resistance among adults, including use of medical devices, previous antibiotic exposure, and ICU admission [14–16]. However, data regarding risk factors among children remain limited. Thus, the purpose of this study was to evaluate risk factors associated with CRKP BSI and mortality of *K. pneumoniae* BSI among paediatric patients as well as to describe clinical characteristics of *K. pneumoniae* BSI.

## Methods

### Study design and patients

This study was conducted at Beijing Children’s Hospital (a 970-bed tertiary paediatric hospital in China with an average of 70,000 admissions per year) between January 2011 and December 2014. Patients (from birth to 18 years of age) with confirmed *K. pneumoniae* BSI were included. A *K. pneumoniae* BSI was defined as the presence of at least once positive blood culture with concomitant signs and symptoms of infection according to established criteria [17, 18]. *K. pneumoniae* BSI cases were identified from the microbiology laboratory database. Only the first episode of *K. pneumoniae* BSI was included. Patients with polymicrobial BSIs or whose medical records were incomplete were excluded. CRKP was defined as minimal inhibition concentration (MIC) for imipenem or meropenem ≥ 4 μg/mL, or isolates were confirmed to produce carbapenemase (https://www.cdc.gov/hai/pdfs/cre/cre-guidance-508.pdf). To assess risk factors for CRKP BSI, a retrospective case-control study was performed. The CRKP group (case group) consisted of patients with CRKP BSI. Patients with BSI due to carbapenem-susceptible *K. pneumoniae* (CSKP) were defined as the CSKP group (control group).

The study was approved by the Ethics Committee of Beijing Children’s Hospital, Capital Medical University. Informed consent was waived because this study did not cause injury to patients.

### Data collection and definitions

The data collected included information regarding demographics, underlying diseases, hospitalization, intensive care unit (ICU) admission, antibiotic therapy, intravascular catheter use, bacterial infections, and immunosuppressive therapy in the 90 days prior to the date of BSI onset; length of hospitalization, microbiological data, antimicrobial therapies, and patient outcomes were also collected.

BSI onset was defined as the collection date of a positive blood culture. BSI was classified as healthcare-related (HCR) or community-acquired (CA). Positive blood cultures obtained from patients who were hospitalized ≥ 48 h, or positive blood cultures obtained from patients who were hospitalized < 48 h but had been hospitalized in the previous six months, were defined as HCR-BSI; CA-BSI were defined as positive blood cultures obtained from patients who were hospitalized < 48 h without having been hospitalized in the previous six months [17]. Neutropenia was defined as absolute neutrophil count (ANC) lower than 500 cells/mm^3^ [18]. Therapy with one or more antimicrobial drugs within ≤ 24 h from BSI onset was defined as empirical antimicrobial therapy; treatment with antimicrobials after the susceptibility results became available was defined as definitive therapy [17]. The active antibiotic agent was defined as MIC within the susceptible range [19]. Appropriate antimicrobial therapy refers to the administration of the in vitro active agent, while inappropriate therapy was defined as treatment without active drugs [20]. The final outcome was determined as survival and all-cause death at 28 days after the date of BSI onset.

### Microbiological methods

The Vitek 2 system (bioMérieux, Marcy l’Etoile, France) and the Phoenix100 automated system (Becton Dickinson, Spark, MD, USA) were used for isolate identification. The MIC values for tested antimicrobial agents were determined by an automated broth microdilution method (Becton Dickinson, Spark, MD, USA). The results were interpreted according to CLSI criteria (CLSI2014) [20]. For colistin, the results were interpreted in accordance with European Committee on Antimicrobial Susceptibility Testing (EUCAST) clinical breakpoints (version 6.0). Polymerase chain reaction (PCR) testing was performed for detection of carbapenemase genes using a previously described method [21].

### Statistical analysis

Categorical variables were presented as numbers and percentages. Continuous variables were presented as the mean and standard deviation (SD) (normally distributed) or median and interquartile range(IQR) (non-normally distributed). Categorical variables were compared using the chi-square or Fisher’s exact tests. Continuous variables were compared by Student’s t test or Mann–Whitney U test according to their distribution.

For univariate analysis, the results were presented as odds ratios (ORs), 95% confidence intervals (CIs) and *P* values. Significant variables with *P* value of < 0.1 were then selected into a logistic regression model for multivariate analysis to evaluate risk factors for CRKP BSIs and for *K. pneumoniae* BSI-related mortality. The discrimination ability of the logistic regression model was assessed by estimating the area under the receiver operating characteristic (ROC) curve. Calibration of the model was assessed using the Hosmer-Lemeshow test for goodness of fit. Two-tailed *P* value of < 0.05 was considered statistically significant. All statistical analyses were performed with SPSS 17.0 software (IBM Corporation).

## Results

### Characteristics of patients

We identified a total of 161 unique cases of bloodstream infections with *K. pneumoniae* during the study period. After excluding 23 cases (polymicrobial BSIs [*n* = 14]; incomplete medical records [*n* = 9]), 138 patients with *K. pneumoniae* BSIs fulfilled the inclusion criteria and were enrolled in this study. Fifty-four cases (39.1%) were identified as CRKP, and 84 (60.9%) were identified as CSKP.

The median patient age was 24.8 months (range, 0 to 204.3 months; interquartile range [IQR], 1.1 to 101.6 months). Patients in the CSKP group were younger than those in CRKP group (10.9 vs. 46.3 months, *P =* 0.021). Eighty (58.0%) were male, and 58 (42.0%) were female. Most of the BSI (114; 82.6%) were healthcare-associated, while the rest (24; 17.4%) were community-acquired. Most children (118; 85.5%) had at least one underlying disease, including hematologic malignancies (66; 55.9%), congenital anomalies (19; 16.1%), premature at birth (13; 11.0%), solid tumours (8; 6.8%), malnutrition (7; 5.9%), and immunodeficiency (6; 5.1%). One hundred and one patients (73.2%) had a hospitalization history within 90 days prior to the onset of BSIs, and the majority of these (69; 68.3%) had been admitted to hematology-oncology department. The median duration of hospitalization was 21 days (IQR, 15 to 32 days), and the median duration of hospital stay before the onset of BSI was 10 days (IQR, 1 to 15 days). The demographic and clinical data of patients with *K. pneumoniae* BSI according to CSKP and CRKP are shown in Table 1.

**Table 1:** Characteristics of 138 patients with KP BSI according to CSKP or CRKP isolates

Variable	Total (*n* = 138)	CSKP (*n* = 84)	CRKP (*n* = 54)	*P*
Age (months)	24.8 (1.1–101.6)	10.9 (0.5–98.9)	46.3 (14.1–107.6)	***0.021***
Male	80 (58.0)	49 (58.3)	31 (57.4)	0.914
Underlying disease	118 (85.5)	67 (79.8)	51 (94.4)	***0.032***
Hematologic malignancies	66 (55.9)	29 (43.3)	37 (72.5)	***0.003***
Congenital anomalies	19 (16.1)	15 (22.4)	4 (7.8)	***0.043***
Prematurity	13 (11.0)	10 (14.9)	3 (5.9)	0.147
Solid tumors	8 (6.8)	6 (9.0)	2 (3.9)	0.463
Malnutrition	7 (5.9)	5 (7.5)	2 (3.9)	0.697
Immunodeficiencies	6 (5.1)	5 (7.5)	1 (2.0)	0.233
Departments				
Hematology-oncology	74 (53.6)	35 (41.7)	39 (72.2)	***< 0.001***
General medical	32 (23.2)	25 (29.8)	7 (13.0)	***0.024***
NICU	14 (10.1)	13 (15.5)	1 (1.9)	***0.009***
PICU	6 (4.3)	2 (2.4)	4 (7.4)	0.210
Surgical	12 (8.7)	9 (10.7)	3 (5.6)	0.366
Healthcare-related infection	114 (82.6)	66 (78.6)	48 (88.9)	0.119
Length of hospitalization	21 (15–32)	21 (15–31.75)	22.5 (12–32.25)	0.825
Length of hospitalization before the onset of BSI	10 (1–15)	8.5 (0–15)	12 (3–15.25)	0.091
Intravascular catheter	108 (78.3)	57 (67.9)	51 (94.4)	***< 0.001***
Mechanical ventilation	17 (12.3)	7 (8.3)	10 (18.5)	0.076
Fever	122 (88.4)	74 (88.1)	48 (88.9)	0.887
Organ disfunction (any)	53 (38.4)	32 (38.1)	21 (38.9)	0.925
Septic shock	15 (10.9)	6 (7.1)	9 (16.7)	0.079
Leukocytes (cells/mm^3^)	690 (200–6170)	4180 (300–6790)	295 (120–3887.5)	***< 0.001***
Neutrophils (cells/mm^3^)	100 (0–2237.5)	935 (0–2960)	10 (0–367.5)	***0.002***
< 500 cells/mm^3^	77 (55.8)	36 (42.9)	41 (75.9)	***< 0.001***
Empirical antibiotic treatment (*n* = 138)				***< 0.001***
No active antibiotic	56 (40.6)	21 (25.0)	35 (64.8)	–
≥ 1 active antibiotic	82 (59.4)	63 (75.0)	19 (35.2)	–
Change in antibiotic treatment after the positive culture	57 (41.3)	29 (34.5)	28 (51.9)	***0.044***
Definitive antibiotic treatment (*n* = 134)				***< 0.001***
No active antibiotic	29 (21.6)	9 (10.8)	20 (39.2)	–
≥ 1 active antibiotic	105 (78.4)	74 (89.1)	31 (60.8)	–
Carbapenem-including treatment(*n* = 134)	95 (70.9)	59 (71.1)	36 (70.6)	0.951
Length of antibiotic treatment	13 (9–18)	14 (9.25–18)	12 (7–18.25)	0.079
Outcome				
7-day mortality	10 (7.2)	1 (1.2)	9 (16.7)	***0.002***
28-day mortality	12 (8.7)	2 (2.4)	10 (18.5)	***0.003***
Hospital mortality	17 (12.3)	7 (8.3)	10 (18.5)	0.076

Data are n (%) or median (IQR)

*P* value in bold italic shows that the variables are statistically significant

### Microbiological characteristics of *K. pneumoniae* strains

**Fig. 1:**
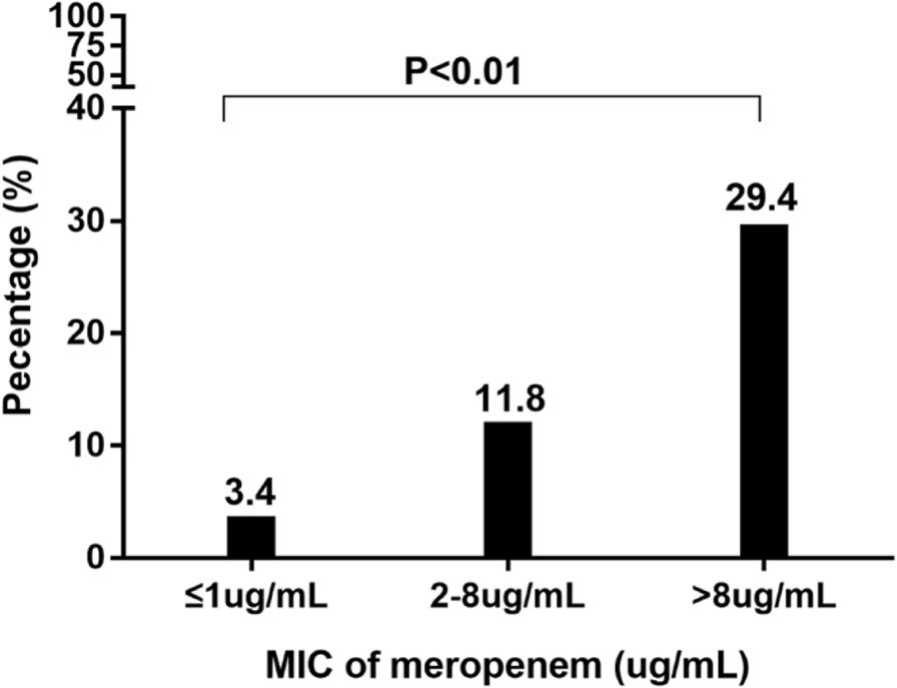
The relationship between mortality and the meropenem MIC of KP isolates

Fifty-four isolates were identified as CRKP. Fifty-one isolates had a meropenem/imipenem MIC ≥ 4 μg/mL. Most of them (40/51, 78.4%) were tested for carbapenemase. The most frequently detected carbapenemase was NDM-1 (23/40, 57.5%) followed by IMP-4 (13/40, 32.5%) and KPC-2 (4/40, 10.0%). Three isolates that were susceptible to meropenem (MIC ≤ 1 μg/mL) and intermediate to imipenem (MIC = 2 μg/mL) were *bla*_IMP-4_ harboring. As shown in Table 2, CRKP isolates showed higher rates of resistance than did CSKP isolates. In the CRKP group, resistance to imipenem and meropenem was 94.4 and 85.2%, respectively. The most active drugs were amikacin (susceptibility of 90.7%, 49/54) and ciprofloxacin (susceptibility of 90.7%, 49/54). MIC of meropenem > 8 μg/ml was associated with higher mortality compared with MIC ≤ 1 μg/ml (Fig. 1).

**Table 2:** Resistance of *K. pneumoniae* strains to specific antimicrobials

Antibiotic (N)	All	CSKP	CRKP	*P* value
Ampicillin (131)	99.2%	100.0%	97.9%	0.359
Ceftazidime (130)	65.4%	47.6%	97.8%	< 0.001
Cefepime (137)	66.4%	45.8%	98.1%	< 0.001
Meropenem (138)	33.3%	0.0%	85.2%	< 0.001
Imipenem (138)	37.0%	0.0%	94.4%	< 0.001
Gentamicin (138)	48.6%	34.5%	70.4%	< 0.001
Amikacin (137)	3.6%	0.0%	9.3%	0.008
Ciprofloxacin (138)	7.2%	6.0%	9.3%	0.465
Trimethoprim-sulfamethoxazole (138)	72.5%	61.9%	88.9%	0.001
Colistin (138)	0.0%	0.0%	0.0%	–

### Risk factors for CRKP BSI

To identify risk factors associated with CRKP BSI, we conducted a retrospective case-control study. On univariate analysis, the following factors were most associated with the development of CRKP BSI: age, underlying disease, hematologic malignancies, number of previous hospitalizations, prior presence of intravascular catheter, previous immunosuppressive therapy, previous neutropenia, previous antibiotic therapy, and number of antibiotic agents. Prior exposure to cephalosporin, antifungal agents and glycopeptides were also significant risk factors.

The results of the multivariate analysis are shown in Table 3: the independent risk factors for CRKP BSI were hematologic malignancies (OR:4.712, [95% CI: 2.181–10.180], *P* <  0.001) and previous cephalosporin administration (OR: 3.427, [95%CI: 1.513–7.766], *P* = 0.003). The result of Hosmer-Lemeshow chi-square testing (X^2^ = 0.588; *P* = 0.745) was indicative of good calibration. The ROC area under the curve was 0.729, suggesting that the multivariate model had good predictive ability.

**Table 3:** Univariate and multi-variate analysis of risk factor for BSI caused by CRKP compared with patients with BSI caused by CSKP

Variable	CSKP (*n* = 84)	CRKP (*n* = 54)	Univariate analysis		Multi-variate analysis	
			OR (95% CI)	*P*	OR (95% CI)	
Age (months)	10.9 (0.5–98.9)	46.3 (14.1–107.6)	–	0.021	–	0.067
Male	49 (58.3)	31 (57.4)	–	0.914	–	–
Underlying disease	67 (79.8)	51 (60.7)	–	0.032	–	0.873
Hematologic malignancies	29 (43.3)	37 (72.5)	4.128 (1.990–8.561)	< 0.001	4.712 (2.181–10.180)	***< 0.001***
Congenital anomalies	15 (22.4)	4 (7.8)	–	0.137	–	–
Prematurity	10 (14.9)	3 (5.9)	–	0.343	–	–
Solid tumors	6 (9.0)	2 (3.9)	–	0.638	–	–
Malnutrition	5 (7.5)	2 (3.9)	–	0.849	–	–
Immunodeficiencies	5 (7.5)	1 (2)	–	0.468	–	–
Prior hospitalization	58 (69.0)	43 (79.6)	–	0.171	–	–
Number of previous hospitalizations	1 (0–4)	3 (1–5)	–	0.023	–	0.873
Previous ICU admission	11 (13.1)	5 (9.3)	–	0.492	–	–
Previous surgery	9 (10.7)	8 (14.8)	–	0.474	–	–
Previous presence of intravascular catheter	41 (48.8)	37 (68.5)	2.283 (1.115–4.671)	0.023	–	0.859
Previous bacterial infections	9 (10.7)	10 (18.5)	–	0.194	–	–
Previous immunosuppressive therapy	33 (39.3)	33 (61.1)	2.429 (1.205–4.894)	0.012	–	0.551
Previous neutropenia	21 (25.0)	30 (55.6)	3.750 (1.808–7.777)	< 0.001	–	0.161
Previous antibiotic therapy	61 (72.6)	49 (90.7)	3.695 (1.309–10.429)	0.006	–	0.067
Number of antibiotic agent	2 (0–3)	3 (2–5)		0.001	–	0.537
Cephalosporins	44 (52.4)	41 (75.9)	2.867 (1.345–6.110)	0.006	3.427 (1.513–7.766)	***0.003***
Penicillins	17 (20.2)	8 (14.8)	–	0.420	–	–
β-Lactam-β-lactamase inhibitor	23 (27.4)	21 (38.9)	–	0.157	–	–
Carbapenems	26 (31)	22 (40.7)	–	0.239	–	–
Aminoglycosides	6 (7.1)	5 (9.3)	–	0.654	–	–
Fluoroquinolones	0 (0.0)	2 (3.7)	–	0.151	–	–
Antifungal agents	22 (26.2)	28 (51.9)	3.035 (1.474–6.249)	0.002	–	0.271
Glycopeptides	24 (28.6)	30 (55.6)	3.125 (1.528–6.392)	0.002	–	0.346
Others	11 (13.1)	10 (18.5)	–	0.387	–	–

Data are n (%) or median (IQR).

*P*-values in bold italic shows variables with evidence of association in univariate and multi-variate analysis

### Treatment and outcome

**Fig. 2:**
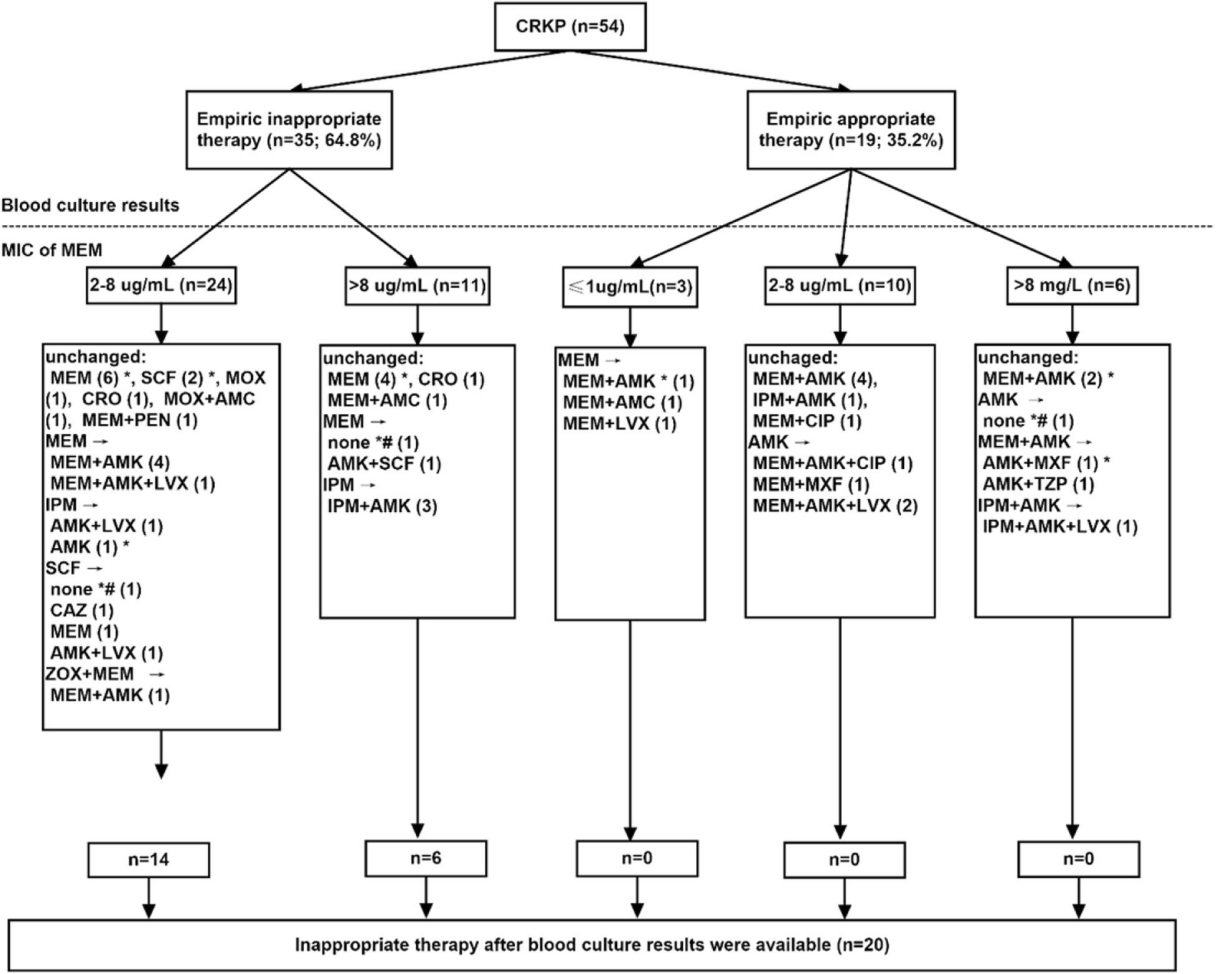
Antibiotic therapy and outcome for patients with CRKP BSIs. AMK, Amikacin; AMC, Amoxycillin/clavulanic acid; CAZ, Ceftazidime; CIP, Ciprofloxacin; CRO, Ceftriaxone; LVX, Levofloxacin; MEM, Meropenem; MOX, Latamoxef; MXF, Moxifloxacin; PEN, Penicillin; SCF, Cefoperazone/sulbactam; TZP, Piperacillin/tazobactam; ZOX, Ceftizoxme. * Died. *# Patients died before blood culture results were available

Antibiotic treatments and outcomes of patients with *K. pneumoniae* BSI are shown in Table 1 and Fig. 2. Patients with CRKP BSI were less likely to receive active antibiotic agents as empirical treatment than were patients with CSKP BSI (19/54 vs. 63/84, *P* < 0.001). Empiric therapy was given to all patients with CRKP BSI: 70.4% (38) received meropenem or imipenem, 18.5% (10) received amikacin, and 16.7% (9) received cephalosporins or β-lactam-β-lactamase inhibitors. Thirty-nine patients received one drug as empiric treatment, while nine received in vitro active drug. Only 27.8% (15) patients received a combination of two or more drugs, while 10 received appropriate empiric therapy. Compared with those of other departments, patients in the hematology-oncology ward received a higher proportion of appropriate empiric therapy (17/39 vs. 2/15, *P* = 0.056). After detection of CRKP BSI, three patients died before antibiotic susceptibility results were available, one of whom had received active antibiotic.

Mortality was significantly higher in patients with CRKP BSI than in those with CSKP BSI (7-day mortality: 16.7% vs. 1.2%, *P* = 0.001; 28-day mortality: 18.5% vs. 2.4%, *P* = 0.003). In the CRKP BSI group, 28-day mortality of patients who received at least one active antibiotic agent as empirical treatment was not significantly different from the mortality of patients who did not receive active antibiotic agent (4/19 vs. 6/35, *P* = 0.728). Carbapenem-including treatment was administered to 39 patients, and their mortality was similar to that of patients who did not receive carbapenem-including treatment (7/39 vs. 3/15, *P* = 1.0). Overall, 28-day mortality was lower among patients in the hematology-oncology ward (6/39, 15.4%) than in patients in other wards (4/15, 26.7%, *P* = 0.438).

Univariate analysis indicated that organ dysfunction, septic shock, mechanical ventilation and isolation of CRKP were associated with 28-day mortality. On multivariate analysis, the independent risk factors for 28-day mortality were mechanical ventilation (OR:9.502, [95% CI: 2.098–43.033], *P* = 0.003), septic shock (OR:6.418, [95% CI: 1.342–30.686], *P* = 0.020), and isolation of CRKP (OR:9.171, [95% CI: 1.546–54.416], *P* = 0.015) (Table 4). The area under the ROC curve for this model was 0.878, and the Hosmer-Lemeshow goodness-of-fit *P*-value was 0.346.

**Table 4:** Univariate and multi-variate analysis of risk factor associated with 28-day mortality among patients with KP BSI

Variable	Survived (*n* = 126)	Died (*n* = 12)	Univariate analysis		Multi-variate analysis	
			OR (95% CI)	*P*	OR (95% CI)	*P*
Age (months), median (IQR)	24.23 (0.91–107.55)	32.41 (3.54–52.99)	–	0.919	–	–
Male	72 (57.1)	8 (66.7)	–	0.739	–	–
Underlying disease	106 (84.1)	12 (100)	–	0.214	–	–
Hematologic malignancies	60 (47.6)	6 (50.0)	–	0.875	–	–
Congenital anomalies	16 (12.7)	3 (25.0)	–	0.457	–	–
Prematurity	12 (9.5)	1 (8.3)	–	1.000	–	–
Malnutrition	6 (4.8)	1 (8.3)	–	1.000	–	–
Immunodeficiencies	6 (4.8)	0 (0)	–	1.000	–	–
Deep venous catheterization	97 (77.0)	11 (91.7)	–	0.417	–	–
Mechanical ventilation	10 (7.9)	7 (58.3)	16.240 (4.352–60.607)	< 0.001	9.502 (2.098–43.033)	***0.003***
Organ dysfunction	44 (34.9)	9 (75.0)	5.591 (1.439–21.718)	0.016	–	–
Septic shock	9 (7.1)	6 (50.0)	13.000 (3.476–48.623)	< 0.001	6.418 (1.342–30.686)	***0.020***
Empirical treatment active in vitro	76 (60.3)	5 (41.7)	–	0.233	–	–
Definitive treatment active in vitro ^a^	100 (79.4)	5 (62.5)	–	0.370	–	–
Carbapenem-including treatment ^a^	90 (71.4)	5 (62.5)	–	0.691	–	–
Isolation of CRKP	44 (34.9)	10 (83.3)	9.318 (1.955–44.421)	0.003	9.171 (1.546–54.416)	***0.015***

^a^ Four patients had died before blood culture results were available.

*P*-values in bold italic shows variables with evidence of association in univariate and multi-variate analysis

## Discussion

CRKP infections are becoming a serious problem in children and are of great concern because of the limited treatment options and unfavourable impact on prognosis [14, 22]. According to previous studies, children with risk factors appear to be more vulnerable to CRKP infections [14]. In the present study, we described the clinical characteristics, risk factors and outcome of BSI due to *K. pneumoniae* in the paediatric population.

In our study, the predominant carbapenemase was NDM-1. In China, the main factor causing carbapenem resistance was KPC-2 among adults [23]; however, NDM-1- producing *K. pneumoniae* was most common in children [21]. Three isolates that were susceptible to meropenem and intermediate to imipenem were *bla*_IMP-4_ harboring, suggesting that some CRKP may test as susceptible or intermediate to carbapenems [14]. Patients with CRKP BSI were older than those with CSKP; this may be because the majority of them came from the hematology-oncology ward.

Previous studies have identified several risk factors associated with development of CRKP BSI, including exposure to healthcare, previous ICU stay or admission, presence of indwelling devices and exposure to antibiotics (such as cephalosporins, fluoroquinolones and carbapenems) [14–16]. We showed that CRKP BSI was associated with several factors including age, underlying disease, number of previous hospitalizations, prior presence of intravascular catheter, previous immunosuppressive therapy, and previous antibiotic therapy. However, only hematologic malignancies and previous cephalosporin administration were independent risk factors for CRKP BSI. Patients with hematologic malignancies usually undergo more frequent exposure to healthcare, longer duration of antibiotic therapy, more invasive procedures and have pre-existing immunosuppression. All of these factors can increase the risk for infections [10, 24]. According to Kwak et al., prior use of carbapenem and cephalosporin were risk factors for acquisition of CRKP [25]. Liu et al. and Orsi et al. found that previous cephalosporin exposure was an independent risk factor ertapenem-resistant *K. pneumoniae* infections [26, 27]. Our study also suggested that previous cephalosporin administration was associated with CRKP BSI. The carbapenems, fluoroquinolones and glycopeptides were independent risk factors for CRKP infections, according to the previous studies [16, 25, 28]; however, the present study did not show an association between these agents and the development of CRKP BSI.

The overall hospital mortality was 12.3%, lower than that reported in previous paediatric studies [29, 30]. The 28-day mortality in the CRKP group (18.5%) was also lower than that of previous reports, where mortality for CRKP BSI ranged from 39 to 82% [31]. Most recently, Montagnani et al. reported that 4 of 9 children (44.4%) in Italy died from CRKP BSI [22]. Another study demonstrated that the rate of carbapenem-resistant Enterobacteriaceae BSI-related mortality in the Indian paediatric population was 52% [32]. Previous studies suggested that ICU admission was an independent risk factor for death [6, 10]; however, compared with this Indian study (38% were in ICU), there were only 9.3% in our cohort. Patients with hematologic malignancies have relatively better baseline condition and undergo less invasive procedures compare with these who admitted to the ICU. All the above factors were the main predictors of CRKP infection mortality [32]. We supposed that these reasons may explain our low mortality rate. On the other hand, 72.5% patients with CRKP BSI had hematologic malignancies in our cohort. Patients with hematologic malignancies often received more effective empirical treatment due to high clinical suspicion for multidrug-resistant gram-negative bacteria in our hematology-oncology ward. It has been shown that appropriate antimicrobial treatment can help to improve the survival rate [32, 33]. Our study also demonstrated that 28-day mortality was lower among patients in the hematology-oncology ward than in patients in other wards.

Consistently with previous studies [10, 33, 34], the mortality appeared to be higher in CRKP BSI than in CSKP BSI (18.5% vs. 2.4%) in our study, and isolation of CRKP was the independent risk factor for 28-day mortality. A review also suggested that the isolation of CRKP was the main risk factor for mortality from BSI [35]. This finding could be explained by the limited CRKP treatment options. Several studies also indicated that empirical therapy with non-active antibiotics may contribute to unfavourable outcomes [36, 37]. In our study, both the proportions of active empirical and definitive antibiotic treatment were significantly lower in patients with CRKP BSI. However, we did not identify an association between active antibiotic agents and mortality. This may be explained by the small number of cases in this cohort.

We also found mechanical ventilation and septic shock were associated with higher 28-day mortality, consistent with the findings of previous studies [38, 39]. Several reports suggested that age and the seriousness of patients’ conditions (including septic shock) were independent risk factors for mortality [16, 31]. Villegas and colleagues also found that critical illness was a statistically significant factor associated with mortality among patients with BSI caused by carbapenemase-producing Enterobacteriaceae [40]. Xu et al. conducted a meta-analysis of mortality of patients infected with *K. pneumoniae* and concluded that patients’ physical condition had a close relationship with their survival [10].

There were some limitations in this study. Firstly, it was a retrospective study and was conducted in a single centre, including 138 paediatric patients. This may have influenced the power of the analysis to identify risk factors. Further prospective, multicentre studies are needed. Secondly, we did not test all the CRKP isolates to determine the carbapenem resistance mechanisms; therefore, it is possible for us to overestimate or underestimate the prevalence of NDM-1.

## Conclusion

Hematologic malignancies and previous cephalosporin administration were associated with the development of CRKP BSI. We also found low mortality caused by *K. pneumoniae* BSI in children. Isolation of CRKP was the independent risk factor for mortality, while patients with serious baseline conditions (including septic shock) had higher mortality. Thus, more attention should be paid to CRKP BSI in the paediatric population, especially in patients with a poor state of health.

## Abbreviations

ANC: Absolute neutrophil count
BSI: Bloodstream infection
CA: Healthcare-related (HCR) or community-acquired
CRKP: Carbapenem-resistant *K. pneumoniae*
CSKP: Carbapenem-susceptible *K. pneumoniae*
ICU: Intensive care unit
MIC: Minimal inhibition concentration
